# Depression and posttraumatic stress disorder in adolescents with nonsuicidal self-injury: comparisons of the psychological correlates and suicidal presentations across diagnostic subgroups

**DOI:** 10.1186/s12888-024-05533-5

**Published:** 2024-02-19

**Authors:** Eunice Seong, Kyung Hwa Lee, Jong-Sun Lee, Sojung Kim, Dong Gi Seo, Jae Hyun Yoo, Doug Hyun Han, Hyunchan Hwang, Chi-Hyun Choi, Jae-Won Kim

**Affiliations:** 1 Division of Child and Adolescent Psychiatry, Department of Psychiatry, Seoul National University Hospital, 101 Daehak-ro, Jongno-gu, Seoul, 03080 Republic of Korea; 2 Department of Psychology, Kangwon National University, 1 Gangwondaehak-gil, Chuncheon, Gangwon-do 24341 Republic of Korea; 3 Department of Psychology, Yeungnam University, 280 Daehak-ro, Gyeongsan, Gyeongbuk 38541 Republic of Korea; 4 Department of Psychology, Hallym University, 1 Hallymdaehak-gil, Chuncheon, Gangwon-do 24252 Republic of Korea; 5 Department of Psychiatry, Seoul St. Mary’s Hospital, College of Medicine, The Catholic University of Korea, 222 Banpo-daero, Seocho-gu, Seoul, 06591 Republic of Korea; 6 Department of Psychiatry, Chung-Ang University College of Medicine, 84 Heukseok-ro, Dongjak-gu, Seoul, 06973 Republic of Korea; 7 Seoul Alpha Neuropsychiatric Clinic, 511 Nonhyeon-ro, Gangnam-gu, Seoul, 06131 Republic of Korea; 8 Department of Psychiatry, Institute of Human Behavioral Medicine, Seoul National University College of Medicine, 103 Daehak-ro, Jongno-gu, Seoul, 03080 Republic of Korea

**Keywords:** Adolescents, Nonsuicidal self-injury, Suicidal cognitions, Suicidal behaviors, Depression

## Abstract

**Background:**

Nonsuicidal self-injury (NSSI) combined with suicide ideation increases the risk of suicidal behaviors. Depression and posttraumatic stress disorder (PTSD) are comorbidities of NSSI compounding this relationship. The present study compared diagnostic subgroups of NSSI based on current depression and PTSD on psychological correlates (i.e., vulnerabilities and impairment) and suicidal presentations (i.e., suicidal cognitions and behaviors) in a psychiatric sample of adolescents.

**Methods:**

Eighty-seven adolescents meeting DSM-5 criteria for NSSI and 104 age-range-matched nonclinical controls (NC) participated. Participants completed self-report measures on psychological vulnerabilities and impairment (e.g., emotion regulation difficulties, negative cognitions). Adolescents with NSSI also completed clinical interviews on psychiatric diagnoses and a recent self-injurious behavior (SIB). Scores on the psychological correlates of NSSI were compared between adolescents with NSSI and NC, and across three diagnostic subgroups of NSSI (A: NSSI+/depression-/PTSD-, *n* = 14; B: NSSI+/depression+/PTSD-, *n* = 57; C: NSSI+/depression+/PTSD+, *n* = 14). Differences between NSSI diagnostic subgroups were tested on the motives for SIB and accompanying suicidal presentations (e.g., desire, intent, motive, lethality).

**Results:**

Common comorbidities of NSSI included depression, panic disorder, generalized anxiety disorder, and PTSD. The NSSI subgroup classification was significantly associated with panic disorder, which was controlled for in the subsequent group comparisons. Overall, adolescents who engage in NSSI with vs. without depression reported more psychological vulnerabilities and impairment and a greater degree of suicidal thoughts/desire in SIB (i.e., groups B, C >A), which remained significant after controlling for panic disorder. An increased odds of the suicidal motive for SIB was found in adolescents with all three conditions (i.e., group C: NSSI+/depression+/PTSD+) compared to those with NSSI but neither depression nor PTSD (i.e., group A: NSSI+/depression-/PTSD-); however, this was not significant after controlling for panic disorder.

**Conclusions:**

Psychological underpinnings of adolescent NSSI in clinical contexts may be largely associated with concurrent depression. Suicidal motives in adolescents who engage in NSSI in the presence of depression and PTSD may be confounded by the co-occurrence of panic disorder. This study warrants the importance of attending to the comorbid depression with NSSI in adolescents as it is related to an increase in suicidal desire accompanying SIB.

**Supplementary Information:**

The online version contains supplementary material available at 10.1186/s12888-024-05533-5.

## Introduction

Nonsuicidal self-injury (NSSI), a deliberate infliction of damage on one’s body without suicidal intent, is a pressing health concern. Importantly, it is a well-documented predictor of suicide attempt [1–3]. NSSI during adolescence represents particular concern not only because it is more prevalent [4, 5] but interrelated with domains that together aggravate the risk of suicidal behaviors, including personality traits (e.g., impulsivity), internalizing symptoms (e.g., depression), and other dysregulated behaviors (e.g., problematic alcohol and/or substance use), as well as later NSSI in adulthood [6–11]. NSSI can occur across an array of psychopathology, including affective and anxiety-related disorders, with common psychological vulnerabilities (e.g., emotion dysregulation, negative cognitions) underpinning these overlaps [12–16]. The overlap of NSSI with other psychiatric disorders and their interrelations contributing to suicide risk suggest that attending to the transdiagnostic nature of NSSI is important for risk formulation among the subgroups with higher vulnerabilities.

A substantial part of the relationship between NSSI and suicidal behaviors has been attributed to the role of suicide ideation (e.g., desire), particularly in the presence of other risk factors (e.g., depression, childhood abuse) [7, 11, 17, 18]. In this regard, the co-occurrence of depression with NSSI merits attention as it is related to an inflation in suicide ideation, on top of NSSI as an indicator of both suicidal desire and the behavioral capability required for lethal behaviors [11, 17, 19, 20]. Despite a heightened risk expected for individuals with comorbid NSSI and depression with regard to suicide-related ideations [21], an explication lacks for this group on specific facets of suicidal cognitions (e.g., passive thoughts/desire/wishes, intent, motive), which are often inconsistently lumped under the construct of suicide ideation [22]. These indices of suicide risk can have varying implications in the progression of ideation to behaviors [23, 24], and understanding and attending to the expressions of suicidal cognitions among individuals who self-injure as distinct yet complementary risk indicators can inform assessment efforts [25, 26].

Another viewpoint to understand the comorbid depression in NSSI can be found in its relation to the motives for NSSI. For example, downregulation of negative emotions, a predominant motivation implicated in the continuation of NSSI, has been highlighted in the context of depressive symptoms and related cognitive processes (e.g., negative attribution, ruminative responses) [27–30]. In addition to the symptom-level approach, depression diagnosis has also been explored in isolation or combined with other diagnostic features (e.g., posttraumatic stress disorder (PTSD), borderline personality disorder) to explain NSSI motives [31, 32]. Whereas these prior studies examined lifetime NSSI with either current or lifetime depression diagnosis, the implication of NSSI co-occurring with other psychiatric conditions can become more temporally relevant by connecting current diagnostic statuses with recent psychological and motivational states and suicidal presentations. This can also help advance the understanding of the clinical significance of NSSI as a proposed diagnostic entity [33] (for full diagnostic criteria, see Supplementary Material 1) and the transdiagnostic nature relative to its comorbidities.

Additional diagnostic features compounding the suicidal risk in NSSI individuals include PTSD. NSSI occurring in the context of PTSD can be elaborated by PTSD symptomatology related to certain motives for NSSI (e.g., avoidance, feeling generation) and the overlapping psychological vulnerabilities that underlie the prognostic trajectories of NSSI and PTSD (e.g., heightened emotional reactivity and dysregulation) [31, 34–36]. The etiology of PTSD that involves exposure to acutely painful and/or fear-inducing experiences may also inform about an increased suicidal risk in the comorbidity of PTSD with NSSI. For example, the Interpersonal Theory of Suicide [18] proposes that certain types of trauma experiences such as childhood physical or sexual abuse can confer a risk for lethal suicidal behaviors by habituating individuals to physical pain and fear of injury. Also, PTSD, compared to depression, may account for suicidal behaviors via its relationship with both internalizing and externalizing vulnerability dimensions, which together constitute risks for the progression of suicide ideation to behaviors [38, 39]. Despite these bases, however, the scope of pertinent knowledge is substantially limited especially in adolescents. Elucidating whether and to what extent suicidal cognitions and behaviors (e.g., desire, intent, lethality) are emphasized in the co-occurrence of NSSI with vs. without depression and/or PTSD represents a meaningful starting point to identify higher-risk groups [40].

The present study seeks to clarify the significance of the current comorbidity of depression and PTSD with NSSI across psychological vulnerabilities and impairment, and suicidal cognitions (e.g., desire, motive) and behaviors (e.g., lethality) in a clinical adolescent sample. We set out to compare on these domains the diagnostic subgroups of adolescents with NSSI based on the sequential addition of current NSSI, depression, and PTSD (group A: NSSI+/depression-/PTSD-; group B: NSSI+/depression+/PTSD-; group C: NSSI+/depression+/PTSD+). Particularly, to provide the rationale for the explored psychological domains as relevant correlates of NSSI in our sample, adolescents with NSSI will first be compared with age-range-matched nonclinical controls (NC) on psychological vulnerabilities and impairment.

Our a priori hypotheses were as follows. First, we predicted higher mean scores in psychological vulnerabilities and impairment (i.e., depressive symptoms, emotion dysregulation, negative cognitions, rumination, perceived stress) for the subgroups with comorbid NSSI and depression (i.e., groups B, C > A). This hypothesis was indirectly based on the association of cognitive content (e.g., automatic negative thoughts, self-criticism) and response styles (e.g., ruminative) with NSSI [15, 28, 41], which is well established in depression, and the association of emotion regulation constructs with NSSI and internalizing disorders (e.g., depression, PTSD) [42–44]. Thus, we also hypothesized that this link will be supported in our sample by higher mean scores in these domains for adolescents with NSSI compared to the NC. Next, we predicted a greater degree (or odds) of suicidal thoughts/desire and motive in adolescents’ recent SIB as well as suicide ideation during the current depressive episode for the subgroups with comorbid NSSI and depression (i.e., group B, C > A) considering the evidence regarding suicide ideation related to NSSI and depression. As concerns the aspects of suicidal behaviors, however, we predicted a greater degree of lethality and suicidal intent only in NSSI + depression + PTSD subgroup compared to the other two subgroups with NSSI (i.e., group C > A, B) [18], along with more seriousness and lethality of suicidal behaviors during the current depressive episode, drawing on the additional account expected of PTSD for suicidal behaviors [23].

## Methods

### Participants and procedure

Participants were 87 adolescents (16.1% boys; 15.3 ± 1.6 years) meeting DSM-5 criteria for NSSI and 104 age-range-matched nonclinical controls (NC, 43.3% boys). Adolescents with NSSI (also referred to as the NSSI group) were referred from four psychiatric clinics and a local mental health care and social service agency in Seoul, South Korea. Inclusion criteria were (1) 6th to 12th graders or adolescents of corresponding ages (ages 11–18); and (2) five or more days of NSSI during the past 12 months (DSM-5 diagnostic criteria for NSSI). The NSSI group completed study procedures including self-report questionnaires on psychological correlates and clinical interviews on psychiatric diagnoses (Kiddie Schedule for Affective Disorders and Schizophrenia Present and Lifetime Version, K-SADS-PL; [45]) and SIB (Suicide Attempt Self-Injury Interview, SASII; [46]). Whereas all participating adolescents in the NSSI group completed questionnaires, one case was omitted from these interviews, resulting in 86 total cases on diagnostic information, suicide-related variables, and features of and motives for SIB.

NC were recruited from online communities and three schools located in Seoul and Gyeonggi district, South Korea. Inclusion criteria were (1) 6th to 12th graders (ages 11–18); and (2) no history of NSSI and other psychiatric diagnoses. Exclusion criteria for both NSSI and NC groups included any diagnoses of autism spectrum disorder and/or intellectual disabilities in the past. NC completed the same questionnaires as the NSSI group but provided them online.

The study received approval from each site’s Institutional Review Board (IRB), with a waiver of documentation of consent from Seoul National University Hospital IRB for the online survey given to NC. Prior to participation, all adolescents in the NSSI group and their parents (or legal guardians) provided written informed consent. NC and their parents (or legal guardians) completed an online consent form according to the approved protocol. Data collection took place from May 2020 to September 2021.

### Psychiatric diagnoses, SIB, and suicidal presentations

The Kiddie Schedule for Affective Disorders and Schizophrenia Present and Lifetime Version (K-SADS-PL) is a semi-structured diagnostic interview used to obtain lifetime and current diagnostic status in children and adolescents based on DSM-5 [45–48]. It previously demonstrated good interrater and test-retest reliability, and concurrent validity for major diagnoses examined in this study, including major depressive disorder, anxiety disorders, and PTSD. Clinical researchers (e.g., master’s-level psychologists) and psychiatrists trained to reliability initially evaluated current and past diagnostic statuses of disorders based on DSM-5 by administering the interview with the parent (or legal guardian) and child, respectively. When necessary, both parties were re-interviewed to resolve informant discrepancies. The average timespan of each interview was between two to four hours. Lifetime diagnosis of a disorder was determined if current and/or past diagnosis was present. Besides diagnostic information, we obtained information on suicide ideation and behaviors during current depressive episodes using four items from the depressive disorders section. Based on a 4-point scale (0=“not at all” to 3=“a lot”), suicide ideation score was obtained by adding up scores on the first two items (i.e., “thoughts of death and/or wish to die” and “thinking about killing oneself with or without a specific plan”), with a higher score indicating greater severity; suicidal behaviors score was likewise obtained by adding up scores on the following two items (i.e., “attempts to kill oneself and wish to die during the attempt” and “seriousness and severity of the attempt”).

The Suicide Attempt Self-Injury Interview (SASII) is a structured interview that assesses multiple aspects of SIB (including suicide attempts), such as the methods used, lethality, probabilities of intervention, actions taken afterwards (e.g., medical treatment), precipitating events, and consequences [46]. It has demonstrated strong test-retest reliabilities and interrater reliabilities, and adequate validity compared to other data sources (e.g., therapist notes, medical records) [46]. Interrater reliability at the item level was also established in an adolescent sample [49]. SASII includes items that describe facets of suicidal cognitions, such as thoughts about suicide and wish to die (suicidal thoughts/desire) and intent to die (suicidal intent), and suicidal behaviors, including medical risk of death (lethality) and patient’s classification of the act as an attempted suicide. In addition to formulating suicide-related variables from these corresponding items (i.e., desire, intent, lethality, suicide attempt), we utilized items on the features of (e.g., methods, impulsivity, dissociation) and reasons/motives for SIB (including a suicidal motive). Each interview took about an hour, and all assessments concerned the most recent episode of SIB.

Suicidal thoughts/desire and impulsivity were reported by the patient in response to the interviewer’s questions and rated on a 7-point scale (from 0 to 6 and 1 to 7, respectively), with higher scores indicating more thoughts/greater desire and greater impulsivity. The item assessed the degree of thinking about suicide or wish to be dead just before or at the time of SIB. Suicidal intent and lethality were rated by the interviewer upon evaluating relevant information on a 5-point (from 1 to 5) and 6-point (from 1 to 6) scale, respectively, with higher scores indicating greater intent and lethality. Suicidal intent was an estimate of the patient’s intent to die and the included seriousness or intensity of the wish and lethality was evaluated based on the method(s) and means that were used for or present at the episode. Dissociation was reported by the patient to indicate whether a feeling of disconnection from oneself or feeling as if one was unreal was present during or prior to SIB.

Assessment of motives was based on the 28 dichotomous items probing the presence of each motive/reason for SIB. Based on Brown et al. (2002) [50] that formerly grouped items on motives into five categories based on an expert consensus, the following categories (with corresponding items) were included: “emotion relief” (seven items), “interpersonal influence” (eight items), “avoidance/escape” (five items), “feeling generation (to feel something or to stop feeling numb)” (two items), and “individual reasons” (six items). However, in this study, we extracted from “individual reasons” two individual categories, “self-punishment” and “to die”, which have been importantly distinguished in literature [51, 52]. Thus, we utilized the first four categories and the two individual categories extracted from the “individual reasons” (i.e., emotion relief, interpersonal influence, avoidance/escape, feeling generation, self-punishment, and to die) that represent the presence (vs. absence) of at least one motive in each category.

### Psychological correlates of NSSI

Center for Epidemiological Studies Depression Scale for Children (CES-DC) is a 20-item self-report scale developed to measure depressive symptoms in children and adolescents. Items are rated on a 4-point scale (0=“not at all” to 3=“a lot”) and the total score is obtained by adding up the scores, with a higher total score indicating more depressive symptoms. The scale demonstrated good internal consistency in prior studies [53–55]. A four-factor structure has been previously replicated with the following subscales: somatic complaints, depressed affect, (lack of) positive affect, and interpersonal problems [53–55]. In addition to the total score, subscale scores were obtained for the present study. The Cronbach’s alpha in the current sample was 0.84 for the total scale and ranged from 0.79 to 0.94 for individual subscales.

The Difficulties in Emotion Regulation Scale (DERS)-16 [56] is a short form of the 36-item original DERS [57]. DERS-16 consists of 16 self-report items that assess the degree of global emotion dysregulation across five domains: non-acceptance of negative emotions (three items), inability to engage in goal-directed behaviors when distressed (three items), difficulties controlling behavioral impulses when distressed (three items), limited access to emotion regulation strategies perceived as effective (five items), and lack of emotional clarity (two items) [56]. Items are rated on a 5-point scale (1=“almost never” to 5=“almost always”), and a higher total score reflects a greater degree of emotion dysregulation. The internal consistency of DERS-16 was excellent in previous studies, with its construct validity comparable to its original full version [56, 58, 59]. The five-factor structure of this scale has been confirmed across samples including psychiatric adolescents [58, 59]. In addition to the total score, subscale scores were obtained for the present study. The Cronbach’s alpha in the current sample was 0.97 for the total scale and ranged from 0.92 to 0.94 for individual subscales.

The Automatic Thoughts Questionnaire (ATQ) [60] is a 30-item self-report scale that measures negative cognitions and self-statements associated with depression. Items are rated on a 5-point scale (1=“not at all” to 5=“all the time”), with a higher total score indicating more negative cognitions. The scale demonstrated high internal consistency in prior research (α=0.96) [60, 61] and in the current study (α=0.98).

The Ruminative Response Scale (RRS) is a 22-item self-report scale that assesses ruminative responses related to depressed mood [62, 63]. Items are rated on a 4-point scale (1=“almost never” to 4=“almost always”), and a higher total score indicates more ruminative responses. The scale previously demonstrated high internal consistency [62–64] and exhibited a three-factor structure, with the following subscales: brooding, reflective pondering, and depressive rumination [64–66]. Because the item composition of subscales differed across studies, we calculated subscale scores based on prior findings from a Korean adolescent sample [64]. The Cronbach’s alpha in the present sample was 0.96 for the total scale and ranged from 0.85 to 0.93 for individual subscales.

The Perceived Stress Scale-10 (PSS-10) is a 10-item self-report scale that measures the degree to which individuals perceive aspects of life as uncontrollable, unpredictable, and overloading [67]. Items are rated on a 5-point scale (0=“never” to 4=“very often”), and a higher total score indicates more perceived stress. Internal consistency of this scale has been previously supported, and a two-factor structure consisting of perceived helplessness and perceived self-efficacy has been replicated in studies, including adolescent samples [67–69]. In addition to the total score, subscale scores were also obtained for the present study. The Cronbach’s alpha was 0.67 for the total scale and ranged from 0.71 to 0.88 for individual subscales in the present sample.

### Data analysis

We first examined the demographic characteristics of the NSSI group and NC, respectively, and the diagnostic characteristics of the NSSI group (Demographic characteristics of NC are summarized in Supplementary Material 2). 86 adolescents in the NSSI group who completed K-SADS-PL were divided into three subgroups based on current depression and PTSD diagnostic statuses (i.e., group A = NSSI+/depression-/PTSD-; group B = NSSI+/depression+/PTSD-; group C = NSSI+/depression+/PTSD+). One case with PTSD without depression (NSSI+/depression-/PTSD+) was dropped, leaving 85 cases for subgroup analyses. Next, we conducted a series of regression analyses to compare the total and subscale scores on psychological correlates (i.e., depressive symptoms, emotion dysregulation, negative cognitions, rumination, and perceived stress) between the NSSI group and NC, controlling for sex and grade (Data presented in Supplementary Material 3). Sex and age were selected as covariates they are possible confounders of adolescent depression and PTSD [70, 71].

Before testing differences between NSSI diagnostic subgroups across domains, we conducted chi-squared tests to examine the association between subgroup classification (groups A, B, and C) and individual diagnoses. Diagnoses significantly associated with subgroup classification were controlled for in subsequent analyses. Diagnostic subgroup differences were tested in psychological correlates, features of (e.g., number of methods, impulsivity) and motives for SIB (e.g., emotion relief, self-punishment), suicidal presentations (e.g., desire, motive, lethality) in SIB, and severity of suicide ideation and behaviors during current depressive episodes (i.e., recent ideation and behaviors). Chi-squared tests were used to identify whether an association exists between each binary variable (i.e., six SIB motives, dissociation, suicide attempt) and subgroup classification. Odds ratio (OR) with 95% CI was calculated using logistic regression analysis to compare the odds for each of these binary variables across pairs of subgroups (i.e., B vs. A, C vs. A, and C vs. B), controlling for sex and age. A series of ANCOVAs with sex and age as covariates and subsequent post-hoc Tukey’s tests were carried out to compare means on continuous variables across three subgroups. Subgroups were additionally compared, controlling for diagnoses that were significantly associated with subgroup classification, along with sex and age (data not presented in Tables but described in text in the Results section). Given the small sample size and even smaller sizes for the subgroups, post-hoc power was calculated for omnibus multivariate F tests and the average per-pair power of Tukey’s test for multiple comparisons across NSSI subgroups (i.e., in psychological correlates, certain features in SIB, and recent suicide ideation and behaviors). Cases for which outcome scores cannot be calculated due to missing responses were omitted from each analysis. Additionally, three standard deviation outlier rejection tests were applied to screen out extreme values in psychological correlates. All analyses were carried out using R studio version 4.2.0.

## Results

### Demographic and diagnostic characteristics of the NSSI group

Table 1 describes the demographic and diagnostic characteristics of the NSSI group (*n* = 87). The most common current diagnosis with NSSI was depression (82.6%), followed by anxiety disorders (any) (43.0%) and PTSD (17.4%). Lifetime diagnoses followed a similar pattern, with depression being the most common diagnosis (87.2%), followed by anxiety disorders (any) (51.2%) and PTSD (19.8%). Among both current and lifetime anxiety diagnoses, panic disorder was most common (19.8% and 23.3% for current and lifetime, respectively), followed by generalized anxiety disorder (18.6% for both current and lifetime) and social anxiety disorder (12.8% for both current and lifetime).

Table 2 presents current diagnostic statuses besides depression and PTSD across three diagnostic subgroups of the NSSI group (groups A, B, and C; see Data analysis section for diagnostic information). The incidence was highest for anxiety disorders (any) in all three subgroups (Group A 21.4%; Group B 40.4%; Group C 71.4%). Among individual diagnoses, generalized anxiety disorder was most frequent in groups A (14.3%) and B (17.5%), but panic disorder in group C (57.1%). The association of the individual diagnoses with NSSI subgroup classification (groups A, B, and C) was significant for panic disorder (*p <.001*).

**Table 1:** Demographic and diagnostic characteristics of the NSSI group (*n* = 87)

	n (%)	
Sex		
Male	14 (16.1%)	
Female	73 (83.9%)	
Age		
12	7 (8.0%)	
13	6 (6.9%)	
14	14 (16.1%)	
15	16 (18.4%)	
16	20 (23.0%)	
17	21 (24.1%)	
18	3 (3.4%)	
Current and lifetime psychiatric diagnoses^a^		
	Current	Lifetime
	n (%)	n (%)
Depressive disorder	71 (82.6%)	75 (87.2%)
Anxiety disorders (any)	37 (43.0%)	44 (51.2%)
Panic disorder	17 (19.8%)	20 (23.3%)
Agoraphobia	4 (4.7%)	5 (5.8%)
Generalized anxiety disorder	16 (18.6%)	16 (18.6%)
Specific Phobia	5 (5.8%)	5 (5.8%)
Social anxiety disorder	11 (12.8%)	11 (12.8%)
Separation anxiety disorder	4 (4.7%)	10 (11.6%)
Posttraumatic stress disorder	15 (17.4%)	17 (19.8%)
Obsessive compulsive disorder	5 (5.8%)	5 (5.8%)
Attention deficit hyperactivity disorder	7 (8.1%)	8 (9.3%)
Oppositional defiant disorder	3 (3.5%)	6 (7.0%)
Conduct disorder	0 (0.0%)	1 (1.2%)
Anorexia nervosa	1 (1.2%)	1 (1.2%)
Bulimia nervosa	5 (5.8%)	6 (7.0%)

Note. ^a^*n*=86

**Table 2:** Current psychiatric diagnoses in NSSI diagnostic subgroups (*n* = 85)

	A. NSSI+/ depression-/PTSD- (*n* = 14)	B. NSSI+/ depression+/PTSD- (*n* = 57)	C. NSSI+/ depression+/PTSD+ (*n* = 14)	χ²
Anxiety disorders (any)	3 (21.4%)	23 (40.4%)	10 (71.4%)	7.45*
Panic disorder^a^	0 (0.0%)	9 (15.8%)	8 (57.1%)	16.20***
Agoraphobia^a^	0 (0.0%)	3 (5.3%)	1 (7.1%)	0.92
Generalized anxiety disorder^a^	2 (14.3%)	10 (17.5%)	4 (28.6%)	1.12
Specific Phobia^a^	1 (7.1%)	4 (7.0%)	0 (0.0%)	1.05
Social anxiety disorder^a^	1 (7.1%)	8 (14.0%)	1 (7.1%)	0.86
Separation anxiety disorder^a^	0 (0.0%)	2 (3.5%)	2 (14.3%)	3.74
Obsessive compulsive disorder^a^	0 (0.0%)	4 (7.0%)	1 (7.1%)	1.05
Attention deficit hyperactivity disorder^a^	1 (7.1%)	5 (8.8%)	1 (7.1%)	0.07
Oppositional defiant disorder^a^	0 (0.0%)	2 (3.5%)	1 (7.1%)	1.05
Conduct disorder	0 (0.0%)	0 (0.0%)	0 (0.0%)	-
Anorexia nervosa^a^	0 (0.0%)	1 (1.8%)	0 (0.0%)	0.50
Bulimia nervosa^a^	1 (7.1%)	2 (3.5%)	2 (14.3%)	2.41

*Notes.* *p <.05; ***p <.001

^a^Chi-squared approximation with simulated p-value was used (based on 2,000 replications)

### Comparisons of the psychological correlates across diagnostic subgroups of NSSI

Table 3 presents descriptive statistics on the psychological correlates of NSSI. Table 4 compares the mean total and subscale scores on these correlates between the three NSSI subgroups. A series of ANCOVAs with post-hoc Tukey’s test with sex and age as covariates revealed significantly higher mean total scores on all five measures for the subgroups with depression compared to the other (i.e., B, C > A; all *ps < 0.001*). Given that the diagnosis of panic disorder was significantly associated with NSSI subgroup classification, we further tested NSSI subgroup differences after controlling for the diagnosis of panic disorder (data not presented in tables). The differences remained significant after controlling for panic disorder on CES-DC, DERS-16, ATQ, and RRS (i.e., B, C > A; all *ps < 0.001*). However, controlling for panic disorder, the score difference on PSS-10 was significant only between group C and A (i.e., C > A; *p* =.012), and the difference between group B and A persisted at a marginally significant level (i.e., B > A; *p* =.055). Post-hoc power calculation for the omnibus multivariate F tests revealed adequate power (> 80%) across scales, controlling and not controlling for panic disorder. However, the average per-pair power of Tukey’s test for NSSI subgroup comparisons was generally weak, ranging from 2.41% (CES-DC) to 14.81% (PSS-10) when controlling for panic disorder and 5.83% (DERS-16) to 31.52% (PSS-10), not controlling for panic disorder.

Subscale score comparisons similarly revealed significantly higher mean scores for the NSSI subgroups with depression on most subscales (i.e., B, C > A; all *p*s < 0.001), with the exception of the reflective pondering of RRS and perceived self-efficacy of PSS-10; the higher mean scores associated with depression persisted when controlling for panic disorder (data not presented). Yet, a higher mean score on the reflective pondering of RRS in group C than A (i.e., C > A; *p* =.014) disappeared after controlling for panic disorder. Power for the omnibus multivariate F test was adequate (> 80%) across subscales except for perceived self-efficacy of PSS-10 (37.99% and 38.29% when controlling and not controlling for panic disorder, respectively). The average per-pair power of Tukey’s test for multiple subgroup comparisons was overall weak across subscales, with power ranging from 2.01% (difficulties controlling impulsive behaviors of DERS-16) to 15.49% (interpersonal difficulties of CES-DC) when controlling for panic disorder and 2.09% (difficulties controlling impulsive behaviors of DERS-16) to 20.61% (perceived self-efficacy of PSS-10), not controlling for panic disorder.

**Table 3:** Psychological correlates of NSSI across NSSI diagnostic subgroups (*n* = 85)

	A. NSSI+/ depression-/PTSD- (*n* = 14)	B. NSSI+/ depression+/PTSD- (*n* = 57)	C. NSSI+/ depression-/PTSD- (*n* = 14)
Psychological correlates (M (SD))			
CES-DC	25.57 (13.18)	46.11 (10.25)	50.07 (7.51)
Somatic complaints	8.64 (4.91)	15.02 (4.29)	16.00 (4.47)
Depressed affect	7.57 (4.40)	16.20 (4.61)	17.93 (3.05)
Lack of positive affect	7.36 (3.67)	10.54 (2.00)	11.07 (1.07)
Interpersonal difficulties	2.00 (1.75)	4.35 (1.71)	5.07 (1.14)
DERS-16	41.31 (16.07)	66.74 (12.87)	71.14 (10.11)
Lack of emotional clarity	5.08 (1.98)	7.56 (2.30)	8.43 (2.21)
Inability to engage in goal directed behaviors	8.46 (2.88)	13.44 (2.50)	14.00 (1.30)
Difficulties controlling impulsive behaviors	7.69 (4.11)	12.35 (3.25)	12.71 (2.87)
Limited access to emotion regulation strategies	12.38 (6.09)	21.16 (4.53)	22.50 (3.88)
Nonacceptance of negative emotions	7.69 (3.45)	12.23 (3.36)	13.50 (3.32)
ATQ	71.07 (25.52)	121.33 (24.35)	134.29 (10.97)
RRS	45.00 (14.94)	65.46 (12.57)	73.69 (12.62)
Brooding	14.79 (4.98)	22.74 (4.58)	25.00 (4.28)
Reflective pondering	11.43 (4.16)	14.40 (4.66)	17.00 (5.34)
Depressive rumination	18.79 (7.35)	28.32 (5.46)	31.69 (4.11)
PSS-10	21.64 (5.54)	26.68 (5.43)	30.50 (5.63)
Perceived helplessness	13.64 (4.94)	17.72 (4.02)	20.21 (4.61)
Perceived self-efficacy	8.00 (3.16)	8.96 (3.35)	10.29 (2.20)

*Notes*. CES-DC: Center for Epidemiological Studies Depression Scale for Children; DERS: Difficulties in Emotion Regulation Scale; ATQ: The Automatic Thoughts Questionnaire; RRS: The Ruminative Response Scale; PSS: Perceived Stress Scale

*n* = 82 for CES-DC, its subscales, and ATQ; *n* = 84 for DERS-16, RRS, and their subscales; *n* = 85 for PSS-10 and its subscales

**Table 4:** ANCOVA and post-hoc group comparisons of psychological correlates of NSSI (*n* = 85)

	F	Comparison	Mean difference	S.E.	95% CI
CES-DC	26.86***	B vs. A	18.48***	3.15	10.98, 25.97
		C vs. A	22.25***	3.91	12.95, 31.56
		C vs. B	3.78	3.09	-3.58, 11.13
Somatic complaints	13.60***	B vs. A	5.88***	1.36	2.66, 9.11
		C vs. A	6.53***	1.68	2.52, 10.53
		C vs. B	0.64	1.33	-2.52, 3.81
Depressed affect	26.62***	B vs. A	7.85***	1.33	4.67, 11.02
		C vs. A	9.58***	1.66	5.64, 13.52
		C vs. B	1.73	1.31	-1.38, 4.84
Lack of positive affect	13.89***	B vs. A	2.66***	0.67	1.06, 4.26
		C vs. A	3.16***	0.83	1.18, 5.15
		C vs. B	0.50	0.66	-1.07, 2.07
Interpersonal difficulties	15.20***	B vs. A	2.09***	0.50	0.90, 3.28
		C vs. A	2.99***	0.62	1.51, 4.47
		C vs. B	0.90	0.49	-0.27, 2.07
DERS-16	23.65***	B vs. A	23.62***	4.13	13.81, 33.43
		C vs. A	27.43***	5.10	15.30, 39.56
		C vs. B	3.81	3.90	-5.46, 13.08
Lack of emotional clarity	8.52***	B vs. A	2.24**	0.72	0.52, 3.95
		C vs. A	3.10**	0.89	0.98, 5.22
		C vs. B	0.86	0.68	-0.76, 2.48
Inability to engage in goal directed behaviors	25.61***	B vs. A	4.69***	0.76	2.88, 6.50
		C vs. A	5.07***	0.94	2.83, 7.31
		C vs. B	0.38	0.72	-1.33, 2.09
Difficulties controlling impulsive behaviors	11.07***	B vs. A	4.46***	1.07	1.91, 7.01
		C vs. A	4.66**	1.33	1.50, 7.81
		C vs. B	0.20	1.01	-2.21, 2.61
Limited access to emotion regulation strategies	21.46***	B vs. A	8.08***	1.48	4.55, 11.61
		C vs. A	9.18***	1.84	4.81, 13.54
		C vs. B	1.10	1.40	-2.24, 4.43
Nonacceptance of negative emotions	11.82***	B vs. A	4.15***	1.09	1.57, 6.73
		C vs. A	5.43***	1.34	2.24, 8.62
		C vs. B	1.28	1.03	-1.16, 3.71
ATQ	34.01***	B vs. A	45.82***	7.09	28.93, 62.70
		C vs. A	58.98***	8.72	38.22, 79.75
		C vs. B	13.17	6.90	-3.25, 29.59
RRS	19.07***	B vs. A	19.04***	3.99	9.54, 28.54
		C vs. A	26.87***	5.09	14.78, 38.97
		C vs. B	7.84	4.04	-1.77, 17.44
Brooding	21.10***	B vs. A	7.33***	1.41	3.98, 10.67
		C vs. A	9.47***	1.79	5.21, 13.73
		C vs. B	2.14	1.42	-1.24, 5.52
Reflective pondering	4.68*	B vs. A	2.87	1.47	-0.62, 6.36
		C vs. A	5.37*	1.87	0.93, 9.82
		C vs. B	2.51	1.48	-1.02, 6.04
Depressive rumination	21.25***	B vs. A	8.85***	1.73	4.74, 12.95
		C vs. A	12.03***	2.20	6.80, 17.26
		C vs. B	3.19	1.75	-0.96, 7.34
PSS-10	9.40***	B vs. A	4.24*	1.68	0.24, 8.25
		C vs. A	8.20***	2.11	3.18, 13.21
		C vs. B	3.95*	1.65	0.03, 7.87
Perceived helplessness	8.66***	B vs. A	3.53*	1.32	0.40, 6.66
		C vs. A	6.05**	1.65	2.13, 9.97
		C vs. B	2.53	1.29	-0.54, 5.59
Perceived self-efficacy	1.83	B vs. A	0.72	0.98	-1.62, 3.06
		C vs. A	2.14	1.23	-0.79, 5.08
		C vs. B	1.43	0.97	-0.87, 3.72

Sex and age are covariates in all comparisons

*Notes*. *p <.05; **p <.01; ***p <.001; adjusted p-values were used for group comparisons

A = NSSI+/depression-/PTSD- (*n* = 14); B = NSSI+/depression+/PTSD- (*n* = 57); C = NSSI+/depression+/PTSD+ (*n* = 14)

CES-DC: Center for Epidemiological Studies Depression Scale for Children; DERS: Difficulties in Emotion Regulation Scale; ATQ: The Automatic Thoughts Questionnaire; RRS: The Ruminative Response Scale; PSS: Perceived Stress Scale

*n* = 82 for CES-DC, its subscales, and ATQ; *n* = 84 for DERS-16, RRS, and their subscales; *n* = 85 for PSS-10 and its subscales

### Motives for and features of recent SIB and suicidal presentations in diagnostic subgroups of NSSI

Tables 5 and 6, and 7 present the motives for (including the suicidal motive) and features of a recent SIB, and suicidal presentations in SIB and current depressive episodes. From the six binary motives for SIB, only the motive to die (i.e., suicidal motive) showed a significant association with the diagnostic group classification (χ²=7.25, *p* =.022; see Table 5). Significant subgroup differences were observed in suicidal thoughts/desire (F = 7.64***, *p* <.001) and lethality (F = 3.79*, *p* =.027; see Table 6). Post-hoc pairwise comparisons revealed higher mean scores on suicide thoughts/wish to die in the NSSI subgroups with depression (i.e., B, C > A; 95% CI 0.38–3.28 and 1.02–4.66), which remained significant after controlling for panic disorder (data not presented), but no subgroup differences in lethality. Likewise, in current depressive episodes, significant group differences were observed in both suicide ideation (i.e., thoughts about death and suicide; recent ideation) (F = 12.64***, *p* <.001) and behaviors (i.e., seriousness and lethality; recent behaviors) (F = 3.50*, *p* =.035; see Table 6). Post-hoc comparisons also revealed higher mean scores on recent ideation severity in depression subgroups compared to the other (i.e., B, C > A; 95% CI 0.77–2.46 and 0.33–2.44), which remained significant after controlling for panic disorder, but no subgroup differences in the seriousness and lethality of recent suicidal behaviors.

Table 7 compares the odds of each SIB motive across three pairs of the NSSI subgroups (i.e., B vs. A; C vs. A; C vs. B) using logistic regression analyses, controlling for sex and age. The NSSI subgroup with comorbid depression and PTSD displayed an increased odds of the motive to die compared to the subgroup with neither depression nor PTSD (i.e., C > A; OR 13.23, 95% CI 1.74-284.86). The result is not robust as the upper confidence limit of OR is excessively large due to a small cell count, and the significance disappeared after controlling for panic disorder (data not presented). No significant group differences were found in any comparisons of the other five motives.

**Table 5:** Motives for and clinical features of SIB and suicidal presentations in NSSI diagnostic subgroups (*n* = 85)

	A. NSSI+/ depression-/PTSD- (*n* = 14)	B. NSSI+/ depression+/PTSD- (*n* = 57)	C. NSSI+/ depression+/PTSD+ (*n* = 14)	χ²
Motives for SIB (n (%))				
Emotion relief^a^	12 (85.7%)	47 (82.5%)	14 (100.0%)	2.85
Interpersonal influence	4 (28.6%))	27 (47.4%)	7 (50.0%)	1.80
Avoidance/escape^a^	7 (50.0%)	40 (70.2%)	10 (71.4%)	2.22
Feeling generation	5 (35.7%)	22 (38.6%)	9 (64.3%)	3.34
Self-punishment	3 (21.4%)	26 (45.6%)	9 (64.3%)	5.26
To die (suicidal motive)^a^	1 (7.1%)	13 (22.8%)	7 (50.0%)	7.25*
Features of SIB (M (SD) or n (%))				
Number of methods	1.43 (0.94)	1.39 (0.70)	1.36 (0.50)	*NA*
Impulsivity	5.36 (1.34)	5.73 (1.46)	5.21 (2.46)	*NA*
Dissociation	3 (21.4%)	20 (37.0%)	9 (64.3%)	5.67
Suicidal cognitions and behaviors (M (SD) or n (%))				
Thoughts/desire	1.00 (1.57)	2.79 (2.05)	3.86 (1.88)	*NA*
Intent	1.50 (0.76)	1.83 (0.99)	2.00 (0.55)	*NA*
Lethality	1.57 (0.51)	1.96 (0.52)	2.00 (0.39)	*NA*
Suicide attempt^a^	1 (7.1%)	7 (12.3%)	2 (14.3%)	0.39
Recent ideation (depressive episode)	3.79 (1.67)	5.50 (1.01)	5.29 (0.99)	*NA*
Recent behaviors (depressive episode)	2.71 (1.07)	3.73 (1.39)	3.79 (1.25)	*NA*

*Notes*. *p <.05

*n* = 81 for lethality; *n* = 82 for dissociation and intent; *n* = 84 for impulsivity, recent ideation, and recent behaviors; *n* = 85 for number of methods, thoughts/desire, and suicide attempt

^a^Chi-squared approximation with simulated p-value was used (based on 2,000 replications)

**Table 6:** ANCOVA and post-hoc group comparisons of clinical features of SIB and suicidal presentations by NSSI diagnostic subgroups (*n* = 85)

	F	Comparison	Mean Difference	S.E.	95% CI
Number of methods	0.04	B vs. A	-0.07	0.22	-0.60, 0.46
		C vs. A	-0.07	0.28	-0.73, 0.60
		C vs. B	0.00	0.22	-0.52, 0.52
Impulsivity	0.72	B vs. A	0.59	0.51	-0.61, 1.79
		C vs. A	-0.01	0.63	-1.52, 1.50
		C vs. B	-0.60	0.50	-1.78, 0.58
Thoughts/desire	7.64***	B vs. A	1.83**	0.61	0.38, 3.28
		C vs. A	2.84**	0.77	1.02, 4.66
		C vs. B	1.01	0.60	-0.41, 2.43
Intent	1.16	B vs. A	0.31	0.28	-0.35, 0.98
		C vs. A	0.52	0.35	-0.31, 1.35
		C vs. B	0.21	0.28	-0.45, 0.86
Lethality	3.79*	B vs. A	0.33	0.15	-0.03, 0.70
		C vs. A	0.35	0.19	-0.10, 0.81
		C vs. B	0.02	0.15	-0.34, 0.38
Recent ideation (depressive episode)	12.64***	B vs. A	1.62***	0.35	0.77, 2.46
		C vs. A	1.39**	0.44	0.33, 2.44
		C vs. B	-0.23	0.35	-1.06, 0.60
Recent behaviors (depressive episode)	3.50*	B vs. A	0.90	0.41	-0.08, 1.88
		C vs. A	1.02	0.52	-0.21, 2.24
		C vs. B	0.11	0.40	-0.85, 1.07

Sex and age are covariates in all comparisons

*Notes*. *p <.05; ***p <.001; adjusted p-values were used for group comparisons

A = NSSI+/depression-/PTSD- (*n* = 14); B = NSSI+/depression+/PTSD- (*n* = 57); C = NSSI+/depression+/PTSD+ (*n* = 14)

*n* = 81 for lethality; *n* = 82 for intent; *n* = 84 for impulsivity, recent ideation, and recent behaviors; *n* = 85 for number of methods, and thoughts/desire

**Table 7:** Comparisons of SIB motives across NSSI diagnostic subgroups (*n* = 85)

	Emotional relief	Interpersonal influence	Avoidance/ escape	Feeling generation	Self-punishment	To die
	OR (95% CI)					
B vs. A^a^	0.90 (0.12, 4.38)	2.23 (0.63, 9.28)	2.03 (0.57, 7.20)	0.98 (0.26, 3.86)	3.04 (0.76, 15.68)	4.20 (0.68, 82.45)
C vs. A^a^	–	2.35 (0.49, 12.56)	1.97 (0.40, 10.61)	2.34 (0.46, 12.94)	5.21 (0.95, 35.28)	13.23* (1.74, 284.86)
C vs. B^b^	–	1.05 (0.31, 3.52)	0.97 (0.27, 4.03)	2.39 (0.68, 9.14)	1.72 (0.48, 6.57)	3.15 (0.90, 11.16)

Sex and age are covariates in all regression models

The dash mark (“-“) indicates that OR approximation is not applicable due to insufficient cell counts

*Notes.* *p <.05

A = NSSI+/depression-/PTSD- (*n* = 14); B = NSSI+/depression+/PTSD- (*n* = 57); C = NSSI+/depression+/PTSD+ (*n* = 14)

^a^Reference group is A

^b^Reference group is B

## Discussion

The present study compared diagnostic subgroups of NSSI in a psychiatric adolescent sample on psychological correlates and suicidal presentations on the basis that the extensive relationship of NSSI to suicidal behaviors can be compounded by the co-occurring depression and PTSD via suicide ideation (e.g., desire) and behavioral capability (e.g., lethality). The findings underscore the wide-ranging impairment associated with concurrent depression in adolescents with NSSI across psychological correlates and the suicidal thoughts/desire present in recent SIB. Differences between adolescents who engage in NSSI with vs. without depression (i.e., groups B, C > A) remained significant for the most part adjusting for panic disorder, the potential diagnostic confounder of the subgroup classification upon which the group comparisons were based, suggesting that the clinical significance of NSSI and depression comorbidity is to some degree robust to the existence of other disorders. Additive impairment in NSSI + depression group with vs. without PTSD (i.e., group C vs. B), however, was overall not supported. An increase in the odds of the suicidal motive for recent SIB associated with depression and PTSD comorbidity (i.e., group C > A) was reduced to non-significance after adjusting for panic disorder, suggesting a possible co-contribution of panic disorder to this particular suicidal presentation identified in recent SIB.

### Diagnostic characteristics of adolescents with NSSI

Common psychiatric comorbidities and their prevalence in adolescents with NSSI were comparably found as in earlier studies [72, 73], with the highest incidence of depressive disorder followed by anxiety-related disorders (e.g., panic disorder, generalized anxiety disorder) and PTSD. The diagnostic overlap between NSSI and depression was particularly emphasized in the current sample. Besides the symptom presentation of depression that renders it relevant to motivations for NSSI such as the downregulation of negative emotions in previous studies [32], the presence of suicidal thoughts and behaviors as a diagnostic criterion of depression may also be highlighting this overlap through its significant correlation with NSSI, such as through the anti-suicide motive [74]. Among other psychiatric diagnoses than depression and PTSD, the incidence of (any) anxiety disorders was considerably high in the presence of depression (i.e., in groups B and C). Aligning with the common comorbidity between depression and anxiety disorders outside the context of NSSI, the observation of this comorbidity merits attention within NSSI population since it is related to a greater suicidal risk in addition to globally more negative outcomes (e.g., poorer psychological functioning, greater symptom severity, worse treatment outcomes) relative to its single counterparts [75, 76].

Another noticeable observation from the diagnostic comorbidities of NSSI concerned the association between the subgroup classification (based on depression and PTSD) and panic disorder, along with a comparatively and considerably high incidence of panic disorder in comorbid NSSI and depression group with vs. without PTSD (group C vs. A, B). Related to this diagnostic overlap between panic disorder and PTSD, a possible shared contribution of panic disorder on group differences that concern PTSD (e.g., increased odds of the suicidal motive in group C than A) was also suggested. Concerning suicidal risk among patients with panic disorder, the episodic yet recurrent exposures to acute physical stimuli in panic attacks seem to make it more relevant than other anxiety disorders to an increased capability for lethal (i.e., suicidal) behaviors, giving rise to an assumption about a higher suicidal risk in the presence of panic disorder with NSSI [12, 37]. Still, empirical evidence is considerably limited in this regard, and further research that addresses the comorbidity of panic disorder with NSSI is warranted. In the context of depression, on the other hand, it has been suggested that certain cognitive (i.e., that one might die) and physical arousal symptoms during panic attacks may interact with suicidal thoughts and desire to confer a greater risk for suicidal behaviors [77]. Likewise, the dynamics between NSSI and its comorbidities such as panic disorder need further elaboration to account for suicidal presentation in the high-risk groups, with an account of specific symptoms interacting with other psychological vulnerabilities.

### Psychological vulnerabilities and impairment associated with depression and PTSD in adolescents with NSSI

In support of the hypothesis about the comorbidity of depression with NSSI, the NSSI + depression subgroups reported increased vulnerabilities and impairment across psychological correlates (i.e., groups B, C > A), which were maintained after controlling for panic disorder. Psychological difficulties associated with current depression coupled with its high comorbidity in adolescents who engage in NSSI suggest that psychological underpinnings of NSSI in clinical contexts may be largely associated with concurrent depression.

On the other hand, more vulnerabilities and impairment associated with additive PTSD to depression were not evident across psychological correlates of NSSI, with differences between the NSSI + depression subgroup with vs. without PTSD (i.e., group C vs. B) being non-significant for the most part. An account of these results not being differentiated by PTSD may concern the internalizing-related nature of the outcome domains that were explored (e.g., negative cognitions, rumination): unlike the co-occurrence of depression with other internalizing comorbidities being largely explained by internalizing dimensions, it has been suggested that PTSD also loads on externalizing dimensions [39, 78], which, in our investigation, could have resulted in a considerable portion of the vulnerability and impairment related to the diagnosis not being explained by the domains explored [79]. Another possibility is that both groups with comorbid NSSI and depression (i.e., groups B and C) may be sharing characteristics that are not manifested as a diagnosis of PTSD but rather in the forms of symptom presentations or features of SIB. For example, the prevalence of the avoidance motive for SIB in the NSSI + depression group without PTSD (i.e., group B) which was comparable to the group with PTSD (i.e., group C) may be indicating that only minimal differences exist in avoidance as an unobserved transdiagnostic construct between these two groups that are distinguished by PTSD diagnosis, characterizing their similarities in relevant domains (e.g., emotion regulation). Among the adolescents who were exposed to traumatic events but were not diagnosed with PTSD, such similarity in the underlying construct could have contributed to reduced symptom-level differences with those diagnosed with PTSD. However, symptom-level investigations will be needed for pertinent information.

### **Suicidal presentations in the diagnostic subgroups of NSSI**

The broad-based impairment among adolescents with comorbid NSSI and depression also emerged in suicidal thoughts/desire in recent SIB. This is a likely outcome considering that the diagnostic criteria for depression involve the presence of suicidal thoughts or behaviors, a notion further corroborated by an increased severity of suicide ideation during current depressive episodes in the two depression subgroups (i.e., groups B, C > A). Beyond this criterion-wise view, however, thoughts about suicide and wish to die emphasized in a recent SIB further underpin the clinical relevance of current depression to suicidal presentations in adolescents with NSSI given that the selection of the SIB episode was solely based on its recency regardless of clinical severity. Thinking about suicide and/or having death wishes may be pervasive for adolescents with depression to be manifest in their SIB and/or intense enough for them to undertake SIB in response to these thoughts and urges (e.g., suicidal or anti-suicidal motive) [74]. Such relevance of current depression provides an avenue for understanding suicidal ideation in adolescents with NSSI and clinical implications for intervention. For example, intervention strategies that target depressive symptoms, particularly those that are closely connected to persistent suicidal thoughts and desire, would prove beneficial considering the scale of problems associated with suicide-related ideation combined with NSSI [19, 80].

On the other hand, an increase in the odds of reporting a suicidal motive (i.e., to die) in the presence of all three conditions relative to NSSI with neither depression nor PTSD (i.e., group C > A) requires cautious interpretation in two regards. The upper bound of CI was excessively large due to small cell counts, indicating that the result is not robust, and the result was reduced to non-significance once comorbid panic disorder was accounted for, suggesting that a greater likelihood of the suicidal motive could have been confounded by the co-occurring panic disorder. Replication of this finding is thus necessary, ideally with a continuous (as opposed to dichotomous) measure of suicidal motives and examining PTSD not obfuscated by other disorders (e.g., in an uncomplicated form). Additional research is also warranted to examine the utility of suicidal motives in explaining behavioral outcomes of SIB (e.g., lethality), on its own or in interaction with other indicators (e.g., intent, methods and means).

### Limitations and conclusion

Several limitations of this study warrant mention for interpretation and implication. First, the exploratory and correlational nature of this study precludes causal inferences between variables. Second, measurement concerns exist in the motives for SIB as the categorization is based on an expert consensus without a factor analysis. Motives for SIB were also measured as a presence-absence dichotomy, so the extent of each motive in subgroups cannot be known and compared in the current study. A related issue concerns an exaggerated upper confidence limit of OR in comparing the odds of suicidal motive across the NSSI subgroups, warranting caution regarding interpretations. Next, the small sample that comprises predominantly female adolescents limits the generalization of the current findings. This imbalance can be considered in future endeavors in the context of a comparatively higher prevalence of anxiety disorders in female than male adolescents [81], along with a higher prevalence of panic disorder in female compared to male suicide attempters [82]. Third, we were not able to establish when the latest self-injury episode explored in SASII took place. Since the timeframe involved in the diagnostic criteria differs for NSSI, depression, and PTSD, this lack of information limits discussion on their interrelationships. Also, due to the high incidence of depression among adolescents with PTSD in the sample, we were not able to parcel out NSSI + PTSD comorbidity from depression (i.e., NSSI + PTSD with vs. without depression). Similarly, the current study was limited in that it does not involve a psychiatric control group (e.g., adolescents with depression or PTSD in the absence of NSSI). Lastly, the study did not examine the diagnosis or symptoms of borderline personality disorder, which implies an overlap with depression and anxiety among individuals with NSSI and accounts for the psychological and motivational variances within this population [32, 83, 84].

In conclusion, our study suggests the significance of depression comorbid with NSSI across suicidal thoughts and psychological impairment in adolescent psychiatric patients. Findings emphasize the importance of attending to comorbid depression with NSSI in this group as it is related to increases in suicidal desire accompanying SIB. Different results on suicidal thoughts/desire as opposed to the intent and motive in this study also necessitate a comprehensive evaluation of suicidal cognitions in suicide risk assessment and warrant future studies to elaborate on their relationship to suicidal behaviors.

### Electronic supplementary material

Below is the link to the electronic supplementary material.

Supplementary Material 1

Supplementary Material 2

Supplementary Material 3

## Data Availability

The datasets used and/or analyzed during the current study are available from the corresponding author upon reasonable request.

## Abbreviations

NSSI: Nonsuicidal self-injury
PTSD: Posttraumatic stress disorder
SIB: Self-injurious behavior
NC: Nonclinical controls

## References

[1] Guan K, Fox KR, Prinstein MJ (2012). Nonsuicidal self-injury as a time-invariant predictor of adolescent suicide ideation and attempts in a diverse community sample. J Consult Clin Psychol.

[2] Ribeiro JD, Franklin JC, Fox KR, Bentley KH, Kleiman EM, Chang BP, Nock MK (2016). Self-injurious thoughts and behaviors as risk factors for future suicide ideation, attempts, and death: a meta-analysis of longitudinal studies. Psychol Med.

[3] Wilkinson P, Kelvin R, Roberts C, Dubicka B, Goodyer I (2011). Clinical and psychosocial predictors of suicide attempts and nonsuicidal self-injury in the adolescent Depression antidepressants and Psychotherapy Trial (ADAPT). Am J Psychiatry.

[4] Plener PL, Schumacher TS, Munz LM, Groschwitz RC (2015). The longitudinal course of non-suicidal self-injury and deliberate self-harm: a systematic review of the literature. Borderline Personality Disorder and Emotion Dysregulation.

[5] Swannell SV, Martin GE, Page A, Hasking P, St John NJ (2014). Prevalence of nonsuicidal self-injury in nonclinical samples: systematic review, meta‐analysis and meta‐regression. Suicide and Life‐Threatening Behavior.

[6] Daukantaitė D, Lundh L-G, Wångby-Lundh M, Claréus B, Bjärehed J, Zhou Y, Liljedahl SI (2021). What happens to young adults who have engaged in self-injurious behavior as adolescents? A 10-year follow-up. Eur Child Adolesc Psychiatry.

[7] Fox KR, Franklin JC, Ribeiro JD, Kleiman EM, Bentley KH, Nock MK (2015). Meta-analysis of risk factors for nonsuicidal self-injury. Clin Psychol Rev.

[8] Mars B, Heron J, Crane C, Hawton K, Kidger J, Lewis G, Macleod J, Tilling K, Gunnell D (2014). Differences in risk factors for self-harm with and without suicidal intent: findings from the ALSPAC cohort. J Affect Disord.

[9] Mars B, Heron J, Klonsky ED, Moran P, O’Connor RC, Tilling K, Wilkinson P, Gunnell D (2019). Predictors of future suicide attempt among adolescents with suicidal thoughts or non-suicidal self-harm: a population-based birth cohort study. The Lancet Psychiatry.

[10] Tuisku V, Kiviruusu O, Pelkonen M, Karlsson L, Strandholm T, Marttunen M (2014). Depressed adolescents as young adults–predictors of suicide attempt and non-suicidal self-injury during an 8-year follow-up. J Affect Disord.

[11] Victor SE, Klonsky ED (2014). Correlates of suicide attempts among self-injurers: a meta-analysis. Clin Psychol Rev.

[12] Bentley KH, Cassiello-Robbins CF, Vittorio L, Sauer-Zavala S, Barlow DH (2015). The association between nonsuicidal self-injury and the emotional disorders: a meta-analytic review. Clin Psychol Rev.

[13] Ehring T, Watkins ER (2008). Repetitive negative thinking as a transdiagnostic process. Int J Cogn Therapy.

[14] Hasking P, Claes L (2020). Transdiagnostic mechanisms involved in nonsuicidal self-injury, risky drinking and disordered eating: Impulsivity, emotion regulation and alexithymia. J Am Coll Health.

[15] Sorgi-Wilson KM, Cheung JC, Ciesinski NK, McCloskey MS. (2022). Cognition and non-suicidal self-injury: exploring relationships with psychological functions. Archives of Suicide Research, 1–17. 10.1080/13811118.2022.2106919 PMC9898468 35924878

[16] Zelkowitz RL, Cole DA (2019). Self-criticism as a transdiagnostic process in nonsuicidal self‐injury and disordered eating: systematic review and meta‐analysis. Suicide and Life‐Threatening Behavior.

[17] Klonsky ED, May AM, Glenn CR (2013). The relationship between nonsuicidal self-injury and attempted suicide: converging evidence from four samples. J Abnorm Psychol.

[18] Van Orden KA, Witte TK, Cukrowicz KC, Braithwaite SR, Selby EA, Joiner TE (2010). The interpersonal theory of suicide. Psychol Rev.

[19] Horwitz AG, Czyz EK, King CA (2015). Predicting future suicide attempts among adolescent and emerging adult psychiatric emergency patients. J Clin Child Adolesc Psychol.

[20] King CA, Grupp-Phelan J, Brent D, Dean JM, Webb M, Bridge JA, Spirito A, Chernick LS, Mahabee‐Gittens EM, Mistry RD (2019). Predicting 3‐month risk for adolescent suicide attempts among pediatric emergency department patients. J Child Psychol Psychiatry.

[21] Silverman MM, Berman AL, Sanddal ND, O’carroll PW, Joiner TE (2007). Rebuilding the tower of Babel: a revised nomenclature for the study of suicide and suicidal behaviors part 2: suicide-related ideations, communications, and behaviors. Suicide and Life‐Threatening Behavior.

[22] Silverman MM, Berman AL, Sanddal ND, O’carroll PW, Joiner TE (2007). Rebuilding the tower of babel: a revised nomenclature for the study of suicide and suicidal behaviors part 1: background, rationale, and methodology. Suicide and Life-Threatening Behavior.

[23] Klonsky ED, May AM, Saffer BY (2016). Suicide, suicide attempts, and suicidal ideation. Annu Rev Clin Psychol.

[24] O’Connor RC (2011). Towards an integrated motivational–volitional model of suicidal behaviour. Int Handb Suicide Prevention: Res Policy Pract.

[25] Hubers A, Moaddine S, Peersmann S, Stijnen T, Van Duijn E, Van der Mast R, Dekkers O, Giltay E (2018). Suicidal ideation and subsequent completed suicide in both psychiatric and non-psychiatric populations: a meta-analysis. Epidemiol Psychiatric Sci.

[26] Silverman MM (2006). The language of suicidology. Suicide and Life-Threatening Behavior.

[27] Barrocas AL, Giletta M, Hankin BL, Prinstein MJ, Abela JR (2015). Nonsuicidal self-injury in adolescence: longitudinal course, trajectories, and intrapersonal predictors. J Abnorm Child Psychol.

[28] Cohen N, Mor N, Henik A (2015). Linking executive control and emotional response: a training procedure to reduce rumination. Clin Psychol Sci.

[29] Taylor J, Peterson CM, Fischer S (2012). Motivations for self-injury, affect, and impulsivity: a comparison of individuals with current self‐injury to individuals with a history of self‐injury. Suicide and Life‐Threatening Behavior.

[30] Xavier A, Pinto-Gouveia J, Cunha M, Dinis A. Longitudinal pathways for the maintenance of non-suicidal self-injury in adolescence: the pernicious blend of depressive symptoms and self-criticism. Child & Youth Care Forum; 2017.

[31] Forbes CN, Tull MT, Richmond JR, Chapman AL, Dixon-Gordon KL, Gratz KL (2019). Motives for nonsuicidal self-injury in individuals with lifetime depressive disorders and posttraumatic stress disorder. J Psychopathol Behav Assess.

[32] Peters EM, John A, Baetz M, Balbuena L (2018). Examining the role of borderline personality traits in the relationship between major depression and nonsuicidal self-injury. Compr Psychiatr.

[33] American Psychiatric Association, D. S. M. T. F., & American Psychiatric Association. Diagnostic and statistical manual of mental disorders: DSM-5 (Vol. 5, No. 5). Washington, DC: American psychiatric association; 2013.

[34] Chapman AL, Gratz KL, Brown MZ (2006). Solving the puzzle of deliberate self-harm: the experiential avoidance model. Behav Res Ther.

[35] Ford JD, Gómez JM (2015). The relationship of psychological trauma and dissociative and posttraumatic stress disorders to nonsuicidal self-injury and suicidality: a review. J Trauma Dissociation.

[36] Smith NB, Kouros CD, Meuret AE (2014). The role of trauma symptoms in nonsuicidal self-injury. Trauma Violence & Abuse.

[37] Silva C, Ribeiro JD, Joiner TE (2015). Mental disorders and thwarted belongingness, perceived burdensomeness, and acquired capability for suicide. Psychiatry Res.

[38] Commisso M, Temcheff C, Orri M, Poirier M, Lau M, Côté S, Vitaro F, Turecki G, Tremblay R, Geoffroy M-C. (2021). Childhood externalizing, internalizing and comorbid problems: distinguishing young adults who think about suicide from those who attempt suicide. Psychological medicine, 1–8. 10.1017/S0033291721002464 34183077

[39] Flory JD, Yehuda R. (2022). Comorbidity between post-traumatic stress disorder and major depressive disorder: alternative explanations and treatment considerations. Dialogues in clinical neuroscience. 10.31887/DCNS.2015.17.2/jflory PMC4518698 26246789

[40] Lento RM, Carson-Wong A, Green JD, AhnAllen CG, Kleespies PM (2019). Is suicidal behavior in mood disorders altered by comorbid PTSD?. Crisis: The Journal of Crisis Intervention and Suicide Prevention.

[41] Cha CB, Wilson KM, Tezanos KM, DiVasto KA, Tolchin GK (2019). Cognition and self-injurious thoughts and behaviors: a systematic review of longitudinal studies. Clin Psychol Rev.

[42] Aldao A, Nolen-Hoeksema S, Schweizer S (2010). Emotion-regulation strategies across psychopathology: a meta-analytic review. Clin Psychol Rev.

[43] Roley ME, Claycomb MA, Contractor AA, Dranger P, Armour C, Elhai JD (2015). The relationship between rumination, PTSD, and depression symptoms. J Affect Disord.

[44] Seligowski AV, Lee DJ, Bardeen JR, Orcutt HK (2015). Emotion regulation and posttraumatic stress symptoms: a meta-analysis. Cogn Behav Ther.

[45] Kaufman J, Birmaher B, Axelson D, Perepletchikova F, Brent D, Ryan N (2016). K-SADS-PL DSM-5.

[46] Linehan MM, Comtois KA, Brown MZ, Heard HL, Wagner A (2006). Suicide attempt Self-Injury interview (SASII): development, reliability, and validity of a scale to assess suicide attempts and intentional self-injury. Psychol Assess.

[47] Kaufman J, Birmaher B, Brent D, Rao U, Flynn C, Moreci P, Williamson D, Ryan N (1997). Schedule for affective disorders and schizophrenia for school-age children-present and lifetime version (K-SADS-PL): initial reliability and validity data. J Am Acad Child Adolesc Psychiatry.

[48] Kim YS, Cheon KA, Kim BN, Chang SA, Yoo HJ, Kim JW, Cho SC, Seo DH, Bae MO, So YK (2004). The reliability and validity of kiddie-schedule for affective disorders and schizophrenia-present and lifetime version-korean version (K-SADS-PL-K). Yonsei Med J.

[49] McCauley E, Berk MS, Asarnow JR, Adrian M, Cohen J, Korslund K, Avina C, Hughes J, Harned M, Gallop R (2018). Efficacy of dialectical behavior therapy for adolescents at high risk for suicide: a randomized clinical trial. JAMA Psychiatry.

[50] Brown MZ, Comtois KA, Linehan MM (2002). Reasons for suicide attempts and nonsuicidal self-injury in women with borderline personality disorder. J Abnorm Psychol.

[51] Klonsky ED (2007). The functions of deliberate self-injury: a review of the evidence. Clin Psychol Rev.

[52] Turner BJ, Chapman AL, Layden BK (2012). Intrapersonal and interpersonal functions of non suicidal self-injury: associations with emotional and social functioning. Suicide and Life‐Threatening Behavior.

[53] Barkmann C, Erhart M, Schulte-Markwort M (2008). The German version of the Centre for Epidemiological Studies Depression Scale for children: psychometric evaluation in a population-based survey of 7 to 17 years old children and adolescents–results of the BELLA study. Eur Child Adolesc Psychiatry.

[54] Essau CA, Olaya B, Pasha G, Gilvarry C, Bray D (2013). Depressive symptoms among children and adolescents in Iran: a confirmatory factor analytic study of the centre for epidemiological studies depression scale for children. Child Psychiatry & Human Development.

[55] Fendrich M, Weissman MM, Warner V (1990). Screening for depressive disorder in children and adolescents: validating the center for epidemiologic studees depression scale for children. Am J Epidemiol.

[56] Bjureberg J, Ljótsson B, Tull MT, Hedman E, Sahlin H, Lundh L-G, Bjärehed J, DiLillo D, Messman-Moore T, Gumpert CH (2016). Development and validation of a brief version of the difficulties in emotion regulation scale: the DERS-16. J Psychopathol Behav Assess.

[57] Gratz KL, Roemer L (2004). Multidimensional assessment of emotion regulation and dysregulation: development, factor structure, and initial validation of the difficulties in emotion regulation scale. J Psychopathol Behav Assess.

[58] Charak R, Byllesby BM, Fowler JC, Sharp C, Elhai JD, Frueh BC (2019). Assessment of the revised difficulties in emotion regulation scales among adolescents and adults with severe mental illness. Psychiatry Res.

[59] Hallion LS, Steinman SA, Tolin DF, Diefenbach GJ (2018). Psychometric properties of the difficulties in emotion regulation scale (DERS) and its short forms in adults with emotional disorders. Front Psychol.

[60] Hollon SD, Kendall PC (1980). Cognitive self-statements in depression: development of an automatic thoughts questionnaire. Cogn Therapy Res.

[61] Kazdin AE (1990). Evaluation of the Automatic thoughts Questionnaire: negative cognitive processes and depression among children. Psychol Assessment: J Consulting Clin Psychol.

[62] Nolen-Hoeksema S (2000). The role of rumination in depressive disorders and mixed anxiety/depressive symptoms. J Abnorm Psychol.

[63] Nolen-Hoeksema S, Morrow J (1991). A prospective study of depression and posttraumatic stress symptoms after a natural disaster: the 1989 Loma Prieta Earthquake. J Personal Soc Psychol.

[64] Shin KM, Cho S-M, Kim K-H (2015). A validation study of the korean-ruminative response scale in Korean adolescents. Psychiatry Invest.

[65] Kim S, Kim JH, Yoon SC (2010). Validation of the korean-ruminative response scale (K-RRS). Korean J Clin Psychol.

[66] Kim S, Kwon J, Yang E, Kim J, Yu B, Lee D (2013). Confirmatory factor analysis of korean-ruminative response scale (K-RRS) in patients with depressive disorders. Cogn Behav Therapy Korea.

[67] Roberti JW, Harrington LN, Storch EA (2006). Further psychometric support for the 10-item version of the perceived stress scale. J Coll Couns.

[68] Liu X, Zhao Y, Li J, Dai J, Wang X, Wang S (2020). Factor structure of the 10-item perceived stress scale and measurement invariance across genders among Chinese adolescents. Front Psychol.

[69] Taylor JM (2015). Psychometric analysis of the ten-item perceived stress scale. Psychol Assess.

[70] Breslau J, Gilman SE, Stein BD, Ruder T, Gmelin T, Miller E (2017). Sex differences in recent first-onset depression in an epidemiological sample of adolescents. Translational Psychiatry.

[71] Garza K, Jovanovic T (2017). Impact of gender on child and adolescent PTSD. Curr Psychiatry Rep.

[72] Auerbach RP, Kim JC, Chango JM, Spiro WJ, Cha C, Gold J, Esterman M, Nock MK (2014). Adolescent nonsuicidal self-injury: examining the role of child abuse, comorbidity, and disinhibition. Psychiatry Res.

[73] Glenn CR, Klonsky ED (2013). Nonsuicidal self-injury disorder: an empirical investigation in adolescent psychiatric patients. J Clin Child Adolesc Psychol.

[74] Czyz E, Glenn CR, Arango A, Koo HJ, King C (2021). Short-term associations between nonsuicidal and suicidal thoughts and behaviors: a daily diary study with high-risk adolescents. J Affect Disord.

[75] Lamers F, van Oppen P, Comijs HC, Smit JH, Spinhoven P, van Balkom AJ, Nolen WA, Zitman FG, Beekman AT, Penninx BW (2011). Comorbidity patterns of anxiety and depressive disorders in a large cohort study: the Netherlands Study of Depression and anxiety (NESDA). J Clin Psychiatry.

[76] Lewinsohn PM, Rohde P, Seeley JR (1995). Adolescent psychopathology: III. The clinical consequences of comorbidity. J Am Acad Child Adolesc Psychiatry.

[77] Katz C, Yaseen ZS, Mojtabai R, Cohen LJ, Galynker II (2011). Panic as an independent risk factor for suicide attempt in depressive illness: findings from the National Epidemiological Survey on Alcohol and related conditions (NESARC). J Clin Psychiatry.

[78] Wolf EJ, Miller MW, Krueger RF, Lyons MJ, Tsuang MT, Koenen KC (2010). Posttraumatic stress disorder and the genetic structure of comorbidity. J Abnorm Psychol.

[79] Poh RY, Zhuang S, Ong XL, Hong RY (2021). Evaluating structural models of cognitive vulnerabilities: Transdiagnostic and specific pathways to internalizing symptoms. Assessment.

[80] Scott LN, Pilkonis PA, Hipwell AE, Keenan K, Stepp SD (2015). Non-suicidal self-injury and suicidal ideation as predictors of suicide attempts in adolescent girls: a multi-wave prospective study. Compr Psychiatr.

[81] Steinsbekk S, Ranum B, Wichstrøm L (2022). Prevalence and course of anxiety disorders and symptoms from preschool to adolescence: a 6-wave community study. J Child Psychol Psychiatry.

[82] Wunderlich U, Bronisch T, Wittchen HU, Carter R (2001). Gender differences in adolescents and young adults with suicidal behaviour. Acta Psychiatrica Scandinavica.

[83] Andover MS, Pepper CM, Ryabchenko KA, Orrico EG, Gibb BE (2005). Self-mutilation and symptoms of depression, anxiety, and borderline personality disorder. Suicide and Life‐Threatening Behavior.

[84] Turner BJ, Dixon-Gordon KL, Austin SB, Rodriguez MA, Rosenthal MZ, Chapman AL (2015). Non-suicidal self-injury with and without borderline personality disorder: differences in self-injury and diagnostic comorbidity. Psychiatry Res.

