# Supplementary material for: Depression and posttraumatic stress disorder in adolescents with nonsuicidal self-injury: comparisons of the psychological correlates and suicidal presentations across diagnostic subgroups
Source: BMC Psychiatry. 2024 Feb 19;24:138. doi: 10.1186/s12888-024-05533-5 (PMC10877746; doi:10.1186/s12888-024-05533-5)
Supplement: Supplementary file 1 — Supplementary Material 1 [file 12888_2024_5533_MOESM1_ESM.docx]

**Supplementary Material 1**

Diagnostic criteria for nonsuicidal self-injury (NSSI) in DSM-5 [84]

A. The individual has engaged in intentional self-inflicted damage (NSSI) on 5 or more days in the past year, with an expectation that it will cause only minor or moderate physical harm (i.e., no suicidal intent).

B. The individual engages in NSSI with one or more of the following expectations:

1) to obtain relief from a negative feeling or cognitive state;

2) to resolve an interpersonal difficulty;

3) to induce a positive feeling state.

C. NSSI is associated with at least one of the following:

1) interpersonal difficulties or negative feelings or thoughts (e.g., depression, anxiety, anger) immediately before NSSI;

2) prior to engaging in NSSI, a period of preoccupation with the intended behavior that is difficult to control;

3) frequent thoughts about self-injury, even when it is not acted upon.

D. The behavior is not socially sanctioned (e.g., body piercing, tattooing) or restricted to picking a scab or nail biting.

E. The behavior or its consequences cause clinically significant distress or interference with functioning in interpersonal, academic, or other important domains.

F. The behavior does not occur exclusively during psychotic episodes, delirium, or substance intoxication or withdrawal. It is not better explained by other psychiatric or medical conditions (e.g., autism spectrum disorder, intellectual disability).
