# Supplementary material for: Depression and posttraumatic stress disorder in adolescents with nonsuicidal self-injury: comparisons of the psychological correlates and suicidal presentations across diagnostic subgroups
Source: BMC Psychiatry. 2024 Feb 19;24:138. doi: 10.1186/s12888-024-05533-5 (PMC10877746; doi:10.1186/s12888-024-05533-5)
Supplement: Supplementary file 2 — Supplementary Material 2 [file 12888_2024_5533_MOESM2_ESM.docx]

**Supplementary Material 2**

Demographic characteristics of nonclinical controls (NC) (n=104)

Of the 104 NC, 43.3% (n=45) were boys. 51.0% (n=53) were 6^th^ graders, 8.7% (n=9) 7^th^ graders, 10.6% (n=11) 8^th^ graders, 5.8% (n=6) 9^th^ graders, 9.6% (n=10) 10^th^ graders, 7.7% (n=8) 11^th^ graders, and 6.7% (n=7) 12^th^ graders.
