# Supplementary material for: Depression and posttraumatic stress disorder in adolescents with nonsuicidal self-injury: comparisons of the psychological correlates and suicidal presentations across diagnostic subgroups
Source: BMC Psychiatry. 2024 Feb 19;24:138. doi: 10.1186/s12888-024-05533-5 (PMC10877746; doi:10.1186/s12888-024-05533-5)
Supplement: Supplementary file 3 — Supplementary Material 3 [file 12888_2024_5533_MOESM3_ESM.docx]

**Supplementary Material 3**

Comparisons of the psychological correlates of nonsuicidal self-injury (NSSI) between adolescents with NSSI (n=87) and nonclinical controls (NC) (n=104)

|  | NSSI (n=87)^a^ | NC (n=104)^b^ | t |
| --- | --- | --- | --- |
|  | M (SD) | M (SD) |  |
| CES-DC | 43.19 (13.04) | 19.11 (12.94) | 9.58*** |
| Somatic complaints | 14.10 (5.02) | 7.08 (4.35) | 7.47*** |
| Depressed affect | 15.02 (5.46) | 6.36 (5.63) | 7.99*** |
| Lack of positive affect | 10.06 (2.54) | 3.94 (3.02) | 11.51*** |
| Interpersonal difficulties | 4.01 (1.91) | 1.73 (1.86) | 6.64*** |
| DERS-16 | 63.41 (15.98) | 34.80 (15.11) | 9.40*** |
| Lack of emotional clarity | 7.30 (2.47) | 4.13 (2.19) | 7.25*** |
| Inability to engage in goal directed behaviors | 12.76 (3.00) | 7.46 (3.33) | 8.32*** |
| Difficulties controlling impulsive behaviors | 11.71 (3.70) | 6.41 (3.19) | 8.24*** |
| Limited access to emotion regulation strategies | 20.00 (5.65) | 10.63 (5.17) | 8.74*** |
| Nonacceptance of negative emotions | 11.64 (3.80) | 6.17 (3.52) | 7.24*** |
| ATQ | 115.06 (30.27) | 62.33 (24.25) | 9.64*** |
| RRS | 63.59 (15.45) | 44.25 (15.68) | 5.72*** |
| Brooding | 21.81 (5.54) | 15.40 (5.54) | 5.41*** |
| Reflective pondering | 14.47 (4.97) | 11.65 (4.65) | 1.60 |
| Depressive rumination | 27.31 (6.80) | 17.19 (6.68) | 7.56*** |
| PSS-10 | 26.53 (5.93) | 18.13 (6.15) | 7.32*** |
| Perceived helplessness | 17.55 (4.63) | 11.13 (5.16) | 6.22*** |
| Perceived self-efficacy | 8.98 (3.17) | 6.99 (3.15) | 4.45*** |

Sex and grade are covariates in all comparisons

*Notes*. ***p < .001

CES-DC: Center for Epidemiological Studies Depression Scale for Children; DERS: Difficulties in Emotion Regulation Scale; ATQ: The Automatic Thoughts Questionnaire; RRS: The Ruminative Response Scale; PSS: Perceived Stress Scale

^a^n=84 for CES-DC, its subscales, and ATQ; n=86 for DERS-16, RRS, and their subscales; n=87 for PSS-10 and its subscales

^b^n=103 for ATQ

The table compares the mean total and subscale scores on the psychological correlates between the NSSI group and NC. A series of regression analyses controlling for sex and grade revealed significantly higher mean total scores on all five measures in the NSSI group than in NC (all *ps<0.001*). Subsequent analyses on the subscale scores remained significant (all *ps<0.001*) except on the reflective pondering subscale of RRS.
